# An Antagonistic Peptide of Gpr1 Ameliorates LPS-Induced Depression through the Hypothalamic-Pituitary-Ovarian Axis

**DOI:** 10.3390/biom11060857

**Published:** 2021-06-09

**Authors:** Rongrong Li, Chiyuan Ma, Yue Xiong, Huashan Zhao, Yali Yang, Li Xue, Baobei Wang, Tianxia Xiao, Jie Chen, Xiaohua Lei, Baohua Ma, Jian Zhang

**Affiliations:** 1 Key Laboratory of Animal Biotechnology College of Veterinary Medicine, Northwest A&F University, Ministry of Agriculture, Yangling 712100, China; rr.li@siat.ac.cn; 2 Center for Energy Metabolism and Reproduction, Shenzhen Institutes of Advanced Technology, Chinese Academy of Sciences, Shenzhen 518055, China; machiyuan1992@163.com (C.M.); xiongyue158@163.com (Y.X.); hs.zhao@siat.ac.cn (H.Z.); yangyl@siat.ac.cn (Y.Y.); xueli@siat.ac.cn (L.X.); bb.wang@siat.ac.cn (B.W.); tx.xiao@siat.ac.cn (T.X.); jie.chen@siat.ac.cn (J.C.)

**Keywords:** Gpr1, depression, depressive-like behavior, ovary, hypothalamic-pituitary-ovarian axis

## Abstract

Depression affects the reproductive axis at the hypothalamus and pituitary levels, which has a significant impact on female fertility. It has been reported that G protein-coupled receptor 1 (Gpr1) mRNA is expressed in both the hypothalamus and ovaries. However, it is unclear whether there is a relationship between Gpr1 and depression, and its role in ovarian function is unknown. Here, the expression of Gpr1 was recorded in the hypothalamus of normal female mice, and co-localized with gonadotrophin-releasing hormone (GnRH) and corticotropin-releasing factor (CRF). We established a depression mouse model to evaluate the antidepressant effect of G5, an antagonistic peptide of Gpr1. The results show that an intraperitoneal injection of G5 improves depressant–like behaviors remarkably, including increased sucrose intake in the sucrose preference test and decreased immobility time in the forced swimming tests. Moreover, G5 treatment increased the release of reproductive hormone and the expression of ovarian gene caused by depression. Together, our findings reveal a link between depression and reproductive diseases through Gpr1 signaling, and suggest antagonistic peptide of Gpr1 as a potential therapeutic application for hormone-modulated depression in women.

## 1. Introduction

Depression is a prevalent psychiatric disorder affecting over 300 million people worldwide in 2015. Globally, it is the leading cause of suicide, resulting in approximately 800,000 deaths annually. Despite decades of research aiming at treatment optimization, up to 30% of patients fail to respond to multiple treatment steps consisting of pharmacological and/or psychotherapeutic interventions [1,2]. At the most fundamental level, the hypothalamic–pituitary–adrenal (HPA) stress axis is driven by corticotropin-releasing hormone (CRH) neurons in the paraventricular nucleus of the hypothalamus (PVN) upon stress activation, releasing CRF into the hypophyseal portal circulation. Finally, glucocorticoids (for example, corticosterone in rodents and cortisol in human) provide critical negative feedback to the HPA axis to ensure the recovery of a homeostatic state. In general, women are twice as likely to be exposed to depression as men, and has been noted that estradiol, known to increase CBG levels and thereby the resting glucocorticoid levels (total), are higher in women than men and could provide a link between gender differences in depression (Darj et al., 1993) [3]. The differences are largely driven by gonadal hormone changes contributing to sex-specific stress responses and vulnerabilities across the lifespan [4,5]. 

Some female reproductive system diseases are often accompanied by depression, especially polycystic ovary syndrome [6,7,8]. It has been reported that depression may also affect the pregnancy rate of women [9,10,11,12,13]. However, there are few reports about this kind of literature. Obviously, female ovarian dysfunction may cause depression, but it is still unclear whether depression impacts female reproductive function as well. G protein-coupled receptor 1 (GPR1) plays a crucial role in the regulation of the non-genomic estrogen effect, which includes seven transmembrane domains [14]. Given the high expression of Gpr1 mRNA in the central nervous system and ovaries [15,16,17], we hypothesized that there is a relationship between Gpr1, depression, and its role in ovarian function.

The aim of the present study was the establishment of a mouse depression model by lipopolysaccharide-induction (LPS) to evaluate the role of Gpr1 signaling in regulating reproductive disorders caused by depression. Taking Gpr1 as the link, Gpr1 antagonist peptide G5 was used to explore the relationship between depression and the reproductive system, and to study the role of Gpr1 in the ovaries of depressed mice, to provide new ideas for the discovery and treatment of related diseases.

## 2. Materials and Methods

### 2.1. Animals

Female Balb/c mice (7–8 weeks old, weighing 18–22 g) were purchased from the Laboratory Animal Center, Institutes of Biomedicine and Health, Chinese Academy of Sciences, China, and were kept under standard animal housing conditions in accordance with the guidelines of the Committee on the Use of Live Animals for Teaching and Research, Shenzhen Institutes of Advanced Technology, Chinese Academy of Sciences.

### 2.2. Drugs

LPS (Sigma-Aldrich, L4516) and fluoxetine hydrochloride (Sigma-Aldrich, F132) were dissolved in sterile saline. G5 is a peptide selected by our laboratory that can be combined with GPR1 [18].

### 2.3. LPS Challenge

Mice were injected with LPS (0.83 mg/kg) or saline intraperitoneally (i.p.), at a volume of 5 mL/kg. This dose of LPS induced depression-like behaviors 24 h after an LPS challenge without reducing locomotor activity [19]. Behavioral tests were performed 24 h after an LPS challenge in the present study. 

### 2.4. Drug Treatments and Experimental Design

The mice were randomly divided into six groups (*n* = 7 each group): control (received normal saline, 10 mL/kg), LPS (received normal saline, 10 mL/kg), LPS-fluoxetine (received fluoxetine 10 mg/kg), and LPS-G5 (received G5 2.25, 22.5, and 225 mg/kg). Different concentrations of G5 were intraperitoneally administered once daily for 7 consecutive days. On the 7th day, mice were intraperitoneally injected with LPS (0.8 mg/kg), 30 min after the last drug administration. Then, 24 h after LPS challenge, a forced swimming test and sucrose preference test were carried out to evaluate the behavioral activity. The animals were sacrificed after behavioral measurements. Blood samples were collected in dry test tubes without coagulant to obtain the serum. The hippocampus and prefrontal cortex (PFC) were immediately removed and stored at −80 °C for biochemical analysis. A group of mice (*n* = 3) treated with the same drug were sacrificed by decapitation four hours after LPS injection, and blood samples were collected in dry test tubes without coagulant to obtain the serum for corticosterone testing.

### 2.5. Behavioral Tests

#### 2.5.1. Open Field Test (OFT)

The OFT was used to assess the spontaneous locomotor activity of the mice. Briefly, each mouse was placed into the center of the experimental apparatus, consisting of a carton (45 × 45 × 35 cm) with its white floor divided into 25 equal-sized squares (9 × 9 cm) and recorded for 5 min by a camera. This apparatus was cleaned with 5% ethyl alcohol between investigations. The number of crossings and rearings was counted by experienced observers blind to the experimental groups [20].

#### 2.5.2. Forced Swim Test (FST)

Mice were placed in a transparent cylinder 19 cm in diameter and 23 cm deep filled with 23–25 °C water, and videotaped for 6 min. Only the last 4 min were scored for four measures: swimming, immobility, climbing, or other. The predominant behavior was recorded every 5 s. For chronic and subchronic fluoxetine studies in BALB/c mice, the mice were pre-exposed to the forced swim test for 6 min 24 h before test day to increase sensitivity for detecting antidepressant behavioral effects [21]. We used a 6 min rather than the traditional 15 min pretest length because we observed fluoxetine’s antidepressant effects with this shorter pretest duration and to avoid further stress to the animals.

#### 2.5.3. Sucrose Preference Test (SPT)

The SPT was employed to evaluate anhedonia (response to reward) [22]. Before testing, all mice were acclimatized to drinking water and 2% sucrose solution for 3 days before LPS administration to establish a baseline sucrose preference for each mouse. Sucrose solution was filled in a drinking bottle having a stopper valve and placed in the home cage of animals. The relative position of bottles was changed daily to avoid the development of a place preference. On the day of testing, mice were deprived of fluid and food for 2 h prior to testing. At the end of the testing, i.e., 48 h post-LPS, fluid content was measured and preference was calculated using the following equation: sucrose preference (%) = sucrose intake/(sucrose intake + water intake) × 100 [23].

### 2.6. Biochemical Analysis

#### 2.6.1. Blood Sampling and Tissue Extraction

After the behavior tests, all mice were sacrificed by decapitation. Blood samples were collected and centrifuged at 3000 rpm for 15 min at 4 °C to obtain serums. Brain tissues were removed and put on water ice immediately, and the hippocampus and mPFC were dissected and weighed. Blood samples and brain tissue were stored at −80 °C before analysis.

#### 2.6.2. Brain-Derived Neurotrophic Factor (BDNF) and Corticosterone (CORT) Level Measurements by Enzyme-Linked Immunosorbent Assays (ELISAs)

Quantitative determination of BDNF level in the hippocampus was performed by using ELISA kits purchased from the USA (R&D Systems, DBNT00, Minneapolis, MN, USA). The minimum detection limit of BDNF was 15.6 pg/mL and intra-assay coefficients of variation were <10%. The concentration of BDNF was expressed as ng/mg of protein. The circulating level of CORT was determined using a corticosterone assay ELISA kit (LDN, ARE-8100, Nordhorn, Germany). Corticosterone concentration was measured according to the manufacturer’s protocol and expressed as ng/mL. All samples and standards were assayed in duplicate.

#### 2.6.3. Immunohistochemistry and Immunofluorescence

Normal mice without any treatment were anaesthetized using 4% chloral hydrate and first perfused with 0.9% saline and then with cold 4% paraformaldehyde, according to the stereotaxic coordinates of the mouse brain. The mouse brain in stereotaxic coordinates, the brain tissue containing the apparent upper part, tuberal part, and pars mammillaris of the hypothalamus were harvested and postfixed in 4% paraformaldehyde for 24 h, processed for embedding in paraffin, and sectioned. IHC (immunohistochemistry) and IF (immunofluorescence) were carried out on 5 μm sections of paraffin-embedded tissue. The primary antibodies used for IHC and IF were mouse GPR1 (clone 043, gift from B A Zabel and E Butcher, Stanford University, Stanford, CA, USA), CRF (ab8901, Abcam, Cambridge, UK), and GnRH (ab16216, Abcam, Cambridge, UK) diluted 1:100 in PBS with 1% BSA. The secondary antibodies were horseradish peroxidase (HRP)-donkey-anti-rat (ab102182, Abcam, Cambridge, UK) to GPR1, Goat-anti-rat (Alexa-568, Invitrogen, A11077, Waltham, MA, USA), Goat-anti-rabbit (Alexa-488, Invitrogen, A11008, Rockford, IL, USA) diluted 1:200 in PBS with 1% BSA. Staining was visualized using a DAB Substrate Kit for peroxidase (Gene Tech, Hyderabad, India), and slides were counterstained with hematoxylin.

#### 2.6.4. RNA Analysis by Quantitative PCR

Ovaries from mice treated with experimental drugs were obtained and stored in a refrigerator at −80 °C. Total RNA from ovary tissues was extracted using RNAiso Plus and subjected to qPCR analysis. RNA samples (0.5 μg) were reverse transcribed into cDNA, according to the manufacturer’s instructions (Toyobo, Osaka, Japan). The PCR mixtures contained 10 μL SYBR Premix Ex Taq II (QSP-101, Toyobo, Japan), 1 μL of each primer, 1 μL cDNA, and 7 μL DNase-free water to a final volume of 20 μL. Cycle conditions were 10 s at 95 °C, followed by 45 cycles at 95 °C for 5 s, at 60 °C for 30 s, and at 72 °C for 30 s. The reaction was completed with a dissociation step for melting point analysis at 50–95 °C (in increments of 0.5 °C for 10 s each). The primers were designed on the basis of the published sequences and are listed in Table 1.

#### 2.6.5. Proinflammatory Cytokines and Hormone Measurements by RIA

Serum from mice treated with experimental drugs was obtained and stored in a refrigerator at −80 °C. Proinflammatory cytokines, estradiol, progesterone, and androgen levels in serum were measured using commercial iodine-125 radioimmunoassay kits (RIA, Beijing North Institute of Biotechnology Co., Ltd, Beijing, China). The sensitivity of the progesterone, estradiol, and androgen RIA assays was 20 ng/mL. The intra-assay error and inter-assay error were <10 and <15%, respectively.

#### 2.6.6. Data and Statistical Analyses

All data are presented as mean ± SEM, and statistical significance was assessed by either one-way ANOVA followed by Fisher’s least significant difference test for post hoc comparisons or the Student’s *t*-test (GraphPad Prism). A *p* value of <0.05 was considered to be statistically significant. 

## 3. Results

### 3.1. Expression of GPR1, GnRH, and CRF in Mouse Hypothalamus

IHC staining showed that GPR1 was expressed at high levels in the hypothalamus in female mice. 

**Figure 1:**
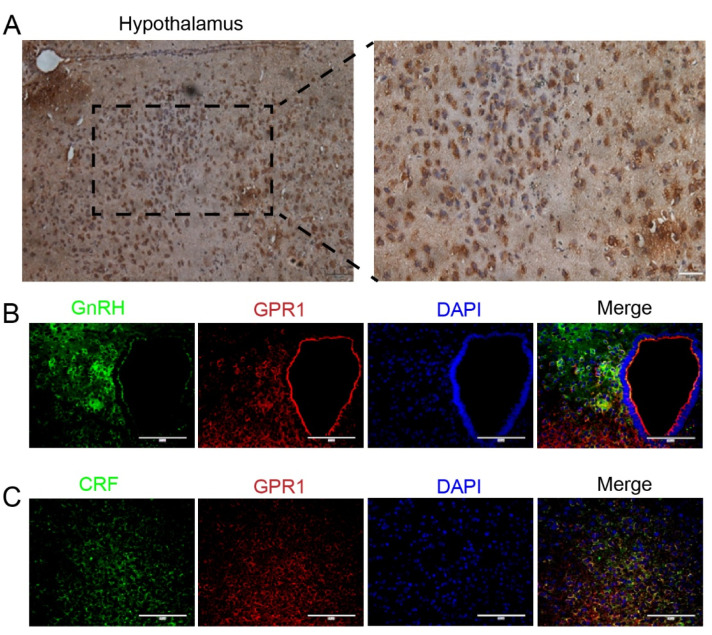
Expression of GPR1 and colocalization of GPR1, GnRH, and CRF in the hypothalamus of female mice. (**A**) Gpr1 expression in the hypothalamus of mice, the brown part of the picture shows GPR1, and the blue part shows a cell nucleus, scale bars represent 50 μm. (**B**) Representative immunostaining images of GnRH and GPR1 in hypothalamus of female mice. (**C**) Representative immunostaining images of CRF and GPR1 in hypothalamus of female mice. Scale bars represent 100 μm.

To further investigate whether GPR1 is related to HPA and hypothalamic–pituitary–ovarian (HPO) axis activity, IHC and IF were used to detect the expression of GPR1, CRF, and GnRH in the hypothalamus of female mice in the same tissue section. As a result, GPR1 was significantly expressed in the cytoplasm of the hypothalamus’s posterior tissue cells in female mice (Figure 1A). More importantly, it can be seen from Figure 1B that GPR1, CRF, and GnRH were both co-localized and expressed in the same cytoplasm. One of the possibilities is that GPR1 may be related to the secretory function of CRF and GnRH in the hypothalamus of female mice.

### 3.2. Effect of GPR1 on Body Weight of LPS-Induced Depression Mice

Subsequently, we established a depression mouse model to detect the antidepressant effect of Gpr1 agonist G5 and the changes of reproductive hormones and related genes. 

**Figure 2:**
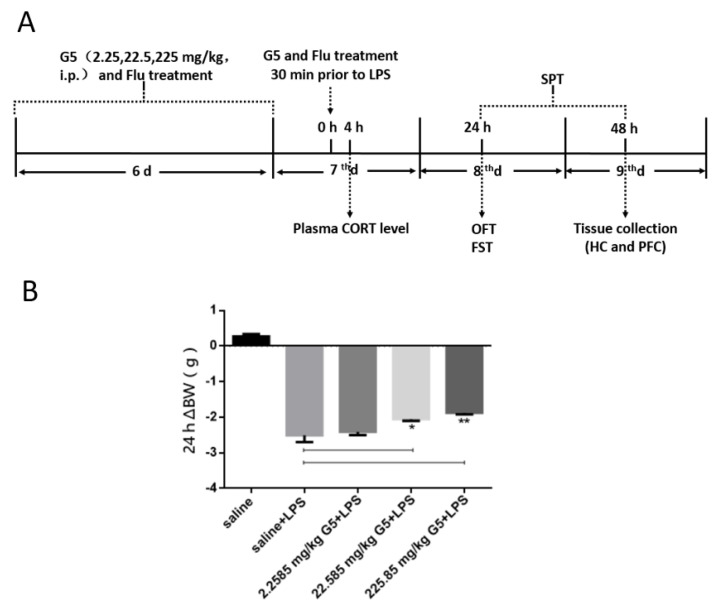
Effect of LPS on body weight of mice. (**A**) Schematic diagram of experimental timeline and study plan. (**B**) Body weight loss at 24 h after LPS challenge; G5 can recover the bodyweight in a dose dependence manner. * *p* < 0.05, ** *p* < 0.01 for one-way ANOVA followed by Fisher’s least significant difference test; *n* = 7, all data are presented as mean ± SEM.

Typically, the peripheral challenge of LPS triggers weight loss, anorexia, and depressive symptoms within 24 h. As illustrated in Figure 2A, body weight was significantly reduced in LPS-challenged mice compared to control mice at 24 h post LPS challenge. By contrast, G5 (2.2585, 22.585, 225.85 mg/kg) treatment conferred significant protective effects against LPS-associated weight loss. G5 effect was dose dependent, and the lowest dose was not effective. (Figure 2B).

### 3.3. Effects of GPR1 on Depression-Like Behaviour 

**Figure 3:**
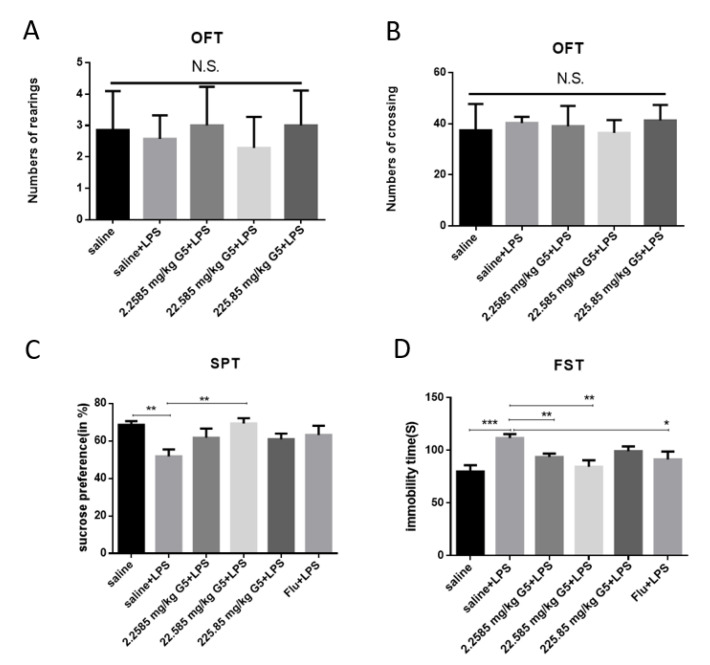
Effects of GPR1 on depression-like behaviors. (**A**,**B**) OFT was assessed before the SPT and FST. (**C**) The SPT was measured 24 h after LPS injection. (**D**) The FST was performed 24 h after LPS injection. For statistical significance, N.S., not significant; * *p* < 0.05, ** *p* < 0.01, *** *p* < 0.005 for one-way ANOVA followed by Fisher’s least significant difference test; *n* = 7, all data are presented as mean ± SEM.

OFT was performed to exclude the possibility of a nonspecific stimulant action of G5, which may create false-positive results for the SPT and FST. As indicated in Figure 3A,B, there were no significant differences in rearing number and movement number among the different groups.

The model LPS groups produced an obvious increase in sucrose consumption compared with the control group (*p* < 0.05). The middle dose of G5–treatment (22.585 mg/kg) also showed a significant improvement in sucrose consumption (*p* < 0.05). The results of the FST are summarized in Figure 3C,D. The immobility time of the LPS group increased obviously compared with the control group (*p* < 0.001). The lower and medium-dose G5 treatment (2.2585 mg/kg and 22.585 mg/kg) significantly decreased immobility in LPS mice (*p* < 0.001). The higher dose DS groups (225.85 mg/kg) showed decreased immobility compared with the LPS group, but the differences were not significant. The Flu groups (10 mg/kg) showed decreased immobility compared with the LPS group, but the differences were also not significant.

### 3.4. Effect of G5 on Inflammatory Factors (TNF-α, IL-6, IL-1β)

**Figure 4:**
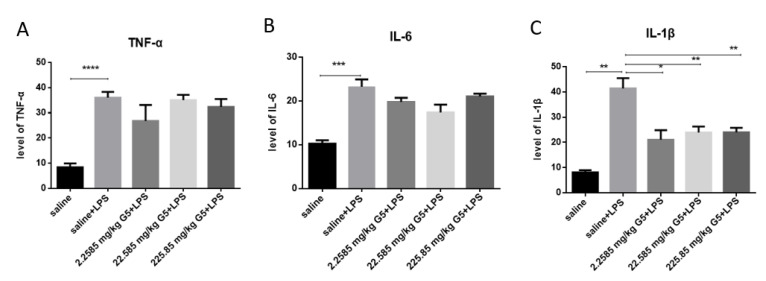
Effect of G5 on Inflammatory factors (TNF-α, IL-6, IL-1β). Mice serum were collected 48 h post-LPS challenge and subjected to RIA kit. The concentrations of (**A**) TNF-α, (**B**) IL-6, and (**C**) IL-1β in serum are presented. For statistical significance, * *p* < 0.05, ** *p* < 0.01, *** *p* < 0.005, **** *p* < 0.001 for one-way ANOVA followed by Fisher’s least significant difference test; *n* = 7, all data are presented as mean ± SEM.

The ELISA method verified the anti-inflammatory effect of G5 in serum. As expected, LPS administration resulted in the induction of all three cytokines in serum (Figure 4A–C). This result further illustrates the success of the LPS-induced depression model. G5 treatment decreased the serum TNF-α, IL-6, and IL-1β levels. Although the levels of inflammatory factors IL-6 and TNF-α had a downward trend, they did not reach statistical significance. It can be seen that the levels of IL-1β decreased in the serum of depression model mice treated with G5, and all concentrations were statistically significant.

### 3.5. Effect of G5 on Level of CORT, BDNF, Progesterone, Estrogen, and Testosterone in Serum in LPS-Induced Depression Mouse Level

**Figure 5:**
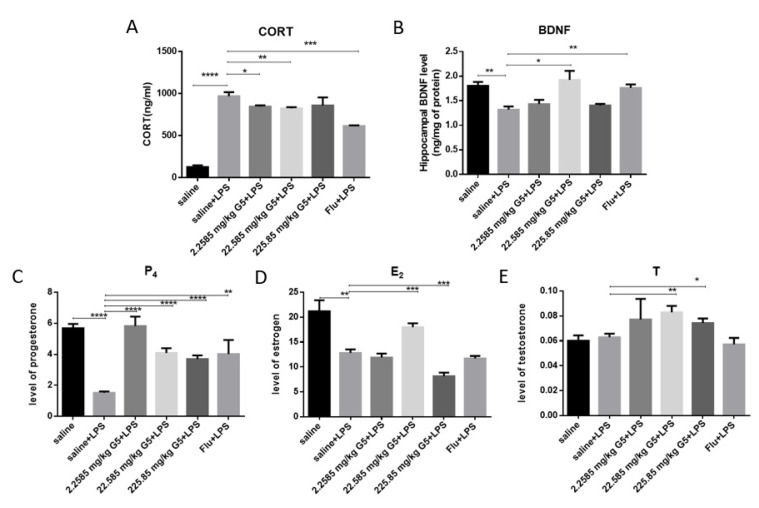
Effect of G5 pre-treatment in LPS challenged animals on serum and level of progesterone (P4), estrogen (E2), and testosterone (T) in LPS-induced depression mice. The concentrations of serum CORT (**A**) and hippocampal BDNF level (**B**). Serum was collected 4 h post-LPS challenge and subjected to ELISA kit to detect CORT. The concentrations of serum P4 (**C**), E2 (**D**), and T level (**E**). Hippocampi and mPFC were collected 48 h post-LPS challenge and subjected to ELISA kit to detect BDNF. Serum were collected 48 h post-LPS challenge and subjected to RIA kit. For statistical significance, * *p* < 0.05, ** *p* < 0.01, *** *p* < 0.005, **** *p* < 0.001 for one-way ANOVA followed by Fisher’s least significant difference test; *n* = 7, all data are presented as mean ± SEM.

LPS significantly (*p* < 0.0001) increased the serum CORT level after 4 h when compared with that of the vehicle-treated group. Pretreatment with G5 (2.2585 and 22.585 mg/kg) significantly (*p* < 0.01) prevented the rise in serum CORT level when compared with that of LPS treated group. Flu pretreatment at the dosages 10 mg/kg (*p* < 0.05) also significantly prevented the elevation of CORT level (Figure 5A). LPS significantly reduced the hippocampal BDNF level (*p* < 0.01) compared with that of the vehicle-treated group. G5 pretreatment at the dosages 22.585 mg/kg (*p* < 0.05) significantly prevented the reduction of BDNF level in HC compared with that of the LPS treated group. Flu pretreatment at the dosages 10 mg/kg (*p* < 0.05) also significantly prevented the reduction of BDNF level (Figure 5B).

On the basis of the above behavioral and molecular test results, the successful establishment of the depression model with LPS is confirmed. In order to determine whether depressed mice will bring about reproductive-related changes, ovaries were taken and reproductive-related hormones were detected to explore the effects of GPR1 on the reproductive aspects of LPS-induced depression through G5. Compared with the normal control group, the levels of progesterone and estrogen in the LPS group were significantly reduced (Figure 5C,D). After G5 pretreatment, the level of progesterone was increased, and depression-like mice treated with a low concentration of G5 reached the same level as the control group. In addition, the level of estrogen also returned to the same level as the control group in depressed mice treated with a medium of concentration G5. However, there was little change in testosterone between the control group and the model group. In addition, G5 alone also increased the level of T (Figure 5E).

### 3.6. Effect of G5 on the Expression of the Star, cyp21a, Aromatase, and 17β-HSD in LPS-Induced Depression Mouse Ovary

**Figure 6:**
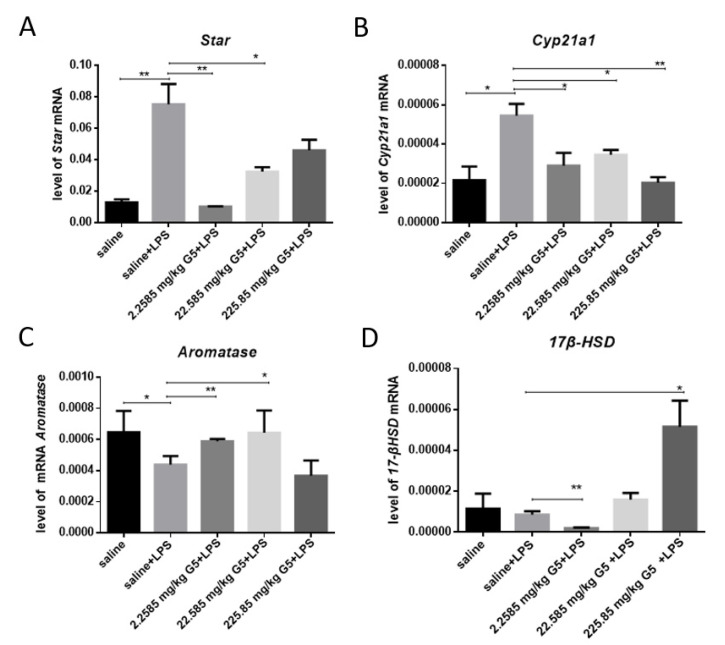
Effect of G5 on the level of gene expression in LPS-induced depression mouse. Ovaries were collected 48 h post-LPS challenge and subjected to mRNA extraction and Q-RT-PCR. Gene expression of (**A**) Star, (**B**) Cyp21a1, (**C**) aromatase, and (**D**) 17β-HSD in hippocampus are presented. For statistical significance, * *p* < 0.05, ** *p* < 0.01 for one-way ANOVA followed by Fisher’s least significant difference test; *n* = 7, all data are presented as mean ± SEM.

The levels of Star and Cyp21a1 in the LPS group significantly increased compared with the normal control group (Figure 6A,B), but the level of estrogen decreased, and G5 pretreatment could reverse these effects. Corresponding to the aromatase changes (Figure 6C), 17β-HSD gene expression levels did not change (Figure 6D).

In addition, we further tested the effect of fluoxetine on the hormones in the serum of depression model mice. Fluoxetine is a selective serum reuptake inhibitor and a classic antidepressant. Its good antidepressant effect has been well verified in changes in BDNF and CORT levels. However, in terms of hormonal changes, Fluoxetine cannot reverse the estrogen changes induced by LPS, and its effect on progesterone is not as obvious as that of G5. Fluoxetine is not the focus of this study, but it shows that due to the special relationship between GPR1 and the ovaries, its antagonistic peptide, G5, can indeed specifically improve the level of reproductive hormones in the serum of LPS-induced depression mice.

## 4. Discussion

Studies on the interaction between the hypothalamus–pituitary–gonad and hypothalamus–pituitary–adrenal gland usually focus on the mechanism of the reproduction ability being disabled by stress. As an important endocrine organ of the female reproductive system, the ovary is closely related to many reproductive diseases. In this study, we measured the levels of ovarian hormone secretion and gene expression in depression-like mice. To the best of our knowledge, we have demonstrated for the first time that the ovarian secretion function of mice with depression-like behavior is significantly impaired; and we also found some differentially expressed genes in the ovaries. In addition, in mice with depression-like behavior, the use of Gpr1 antagonist G5 reversed the changes of depression behavior and reproductive hormones and genes, suggesting that Gpr1 may be the target molecule of these two axis-related diseases.

First, the immunohistochemical staining results in this study confirmed the Gpr1 mRNA expression in the brain, especially in the hypothalamus, which is consistent with a previous report [24]. Then, immunofluorescence staining showed that Gpr1 was co-located with CRH and GnRH in the hypothalamus, suggesting that Gpr1 may play a role in the relationship between depression and reproduction.

The LPS model is a validated and widely adopted behavioral model of depression, and based on the acute activation of the peripheral innate immune system in laboratory animals. LPS challenge and intraperitoneal injection of G5 did not significantly alter the number of rearings and crossings in the center area of the open field in the present study, which demonstrated that the reduction of immobility time in the FST was not influenced by locomotor activity, and thus should be specific to the antidepressant effects [25]. In our study, at 24 h after intraperitoneal injection of LPS, LPS-mice showed a significant decrease in sucrose consumption, consistent with previous reports [26,27], while the administration of G5 reversed this. The FST was performed to reflect behavioral despair [28], and is the most widely employed behavioral test for screening depressive-like behavior and antidepressant efficacy in animals. In the present study, the immobility time of the LPS–expose displayed a significant prolongation in FST, while G5 administration reversed these alterations. The results of the sucrose preference test and forced swimming test support each other. Therefore, the behavioral results showed that the depression mouse model was established successfully.

The role of inflammation in the pathogenesis of depression has been well demonstrated in the literature [29]. To explore the possible mechanism by which G5 exerts its antidepressant activity, we measured serum levels of some inflammatory cytokines. We found that LPS administration induced significant elevation of TNF-α, IL-1β, and IL-6 inflammatory cytokines levels, while these have been reported to contribute to the pathogenesis of depression [29,30,31,32]. This result also implies the success of the mouse model of depression induced by LPS injection. However, G5 pretreatment markedly reduced the level of IL-1β in the low-dose, middle-dose, and high-dose group compared to the LPS group, indicating that G5 may have exerted antidepressant-like activities via IL-1β in the mice.

In order to further verify the depression mouse model at the molecular level, we selected BDNF and CORT as molecular indicators [33,34]. BDNF, a polypeptidic factor, is implicated in the pathophysiology and therapeutic mechanism of depression [35]. Importantly, the distinct alteration of BDNF expression in brain regions of rodents is involved in LPS-induced depression [36,37]. Our present study using ELISA analysis verified that BDNF expression was inhibited in the mPFC and hippocampus in LPS-induced depression in mice. These findings are consistent with reduced BDNF expression in the prefrontal cortex and hippocampus [38]. This change was reversed by a moderate concentration of G5, suggesting that G5 may play an antidepressant role through BDNF. Similarly, we found that LPS increased CORT levels in mice, which was also reversed by G5 pretreatment. These results indicate the efficiency of the established animal model, and the that antagonistic peptide G5 of Gpr1 has a powerful antidepressant effect.

In recent years, some studies have demonstrated that sex steroids contribute to neurogenesis, while the decrease of estrogen or androgens can cause depressive behavior [39,40]. Interestingly, in our research, the levels of progesterone and estrogen were significantly decreased in LPS induced depression mice. Our results may also prove that depression induces abnormal hormone secretion. The process of hormone synthesis is regulated by a series of steroid synthases, including Star, cyp21a1, aromatase, and 17β-HSD. Accordingly, CYP21a1, responsible for progesterone synthesis, and aromatase, which is responsible for androgen synthesis [41], also changed. G5 pretreatment also reversed the changes of reproductive hormones and related genes caused by depression. Indeed, our group previously proved that GPR1 could inhibit the production of P4 in the corpus luteum [17]. After G5 was injected into depression model mice, GPR1 was inhibited by G5, and the level of progesterone increased significantly, suggesting that GPR1 may play a role in the relationship between the nervous system and reproductive system diseases. GPR1 signaling might play an important role via the HPO. This serves as a starting point for future studies on the pathogenesis in comorbid depression and reproductive diseases, and linked by a common target, GPR1. The potential molecular mechanism of GPR1 will be further researched in our next work. 

## 5. Conclusions

This study shed light on ovarian changes in animal models of depression and indicated a strong connection between depression and changes in ovarian function. Inhibitors of Gpr1 have an antidepressant effect and can improve hormone and gene changes caused by depression, suggesting antagonistic peptide of Gpr1 as a potential therapeutic application for hormone-modulated depression in women. 

## Figures and Tables

**Table 1:** Primers for PCR and Real-Time PCR Gene Sequences (5′–3′).

Gene		Sequences (5′–3′)
β-Actin	Forward	GGAAATCGTGCGTGACATTA
	Reverse	AGGAAGGAAGGCTGGAAGAG
Star	Forward	CTGCTAGACCAGCCCATGGAC
	Reverse	TGATTTCCTTGACATTTGGGTTCC
17β-HSD	Forward	AGGACCTGGATTCAGTTCCAAGC
	Reverse	CTAGGTGTTTGAGAAAGTCTG
Aromatase	Forward	GCAATCCTGAAGGAGATCCA
	Reverse	GCCGTCAATTACGTCATCCT
Cyp21a1	Forward	AGGAATTCTCCTTCCTCACTTGT
	Reverse	TCTGTACCAACGTGCTGTCC

## Data Availability

Not applicable.
